# Effect of vitamin D supplementation on cardiac-metabolic risk factors in elderly: a systematic review and meta-analysis of clinical trials

**DOI:** 10.1186/s13098-022-00859-0

**Published:** 2022-06-25

**Authors:** Mostafa Qorbani, Maryam Zarei, Yousef Moradi, Geeta Appannah, Shirin Djalainia, Kumars Pourrostami, Hanieh-Sadat Ejtahed, Armita Mahdavi-Gorabi, Ebrahim Khalil Naderali, Maryam Khazdouz

**Affiliations:** 1 Non-Communicable Diseases Research Center, Alborz University of Medical Sciences, Karaj, Iran; 2 Endocrinology and Metabolism Research Center, Endocrinology and Metabolism Clinical Sciences Institute, Tehran University of Medical Sciences, Tehran, Iran; 3 Department of Nutrition, Faculty of Medicine and Health Sciences, Universiti Putra Malaysia, Serdang, Selangor Malaysia; 4 Social Determinants of Health Research Center, Research Institute for Health Development, Kurdistan University of Medical Sciences, Sanandaj, Iran; 5 Department of Epidemiology and Biostatistics, Faculty of Medicine, Kurdistan University of Medical Sciences, Sanandaj, Iran; 6 Development of Research & Technology Center, Deputy of Research and Technology, Ministry of Health and Medical Education, Tehran, Iran; 7 Obesity and Eating Habits Research Center, Endocrinology and Metabolism Clinical Sciences Institute, Tehran University of Medical Sciences, Tehran, Iran; 8 Social Determinants of Health Research Center, Alborz University of Medical Sciences, Karaj, Iran; 9 Department of Health Sciences, Liverpool Hope University, Hope Park, UK; 10 Growth and Development Research Center, Children’s Medical Center, Tehran University of Medical Sciences, Tehran, Iran; 11 Department of Nutrition Community, Deputy of Health affairs, Ministry of Health and Medical Education, Tehran, Iran

**Keywords:** Vitamin D, Supplementation, Cardiac-metabolic, Elderly

## Abstract

**Background:**

There has been a longstanding interest in the potential effect of vitamin D in preventing cardiac-metabolic diseases. However, there are divergent results regarding the impact of vitamin D supplementation (VDS) on managing cardiac-metabolic outcomes in the elderly population.

**Material and method:**

We systematically searched electronic databases; Web of Science, PubMed, Scopus, EMBASE, Cochrane, and ProQuest. We included all trials that evaluated the effect of VDS on cardiac-metabolic risk factors in the elderly population, which were published until 30 September 2021. The effects of VDS on cardiac-metabolic outcomes were assessed using standardized mean difference (SMD). A random-effect model was used to pool the SMD and 95% confidence interval (CI).

**Result:**

The literature search identified 4409 studies, of which 12 trials met inclusion criteria. Results of random effect meta-analysis indicated a significant reduction in total cholesterol (TC) (SMD: − 0.14 mg/dl; 95% CI: − 0.25, − 0.02) and triglyceride (TG) (SMD: − 0.45 mg/dl; 95% CI: − 0.86, − 0.04) with VDS compared to the placebo. The subgroup analyses revealed that the reduction of TG in patients with diabetes and vitamin D deficiency was significant. Furthermore, short-term intervention (≤ 6 months) induced a significantly lower level of TG and insulin in comparison to longer duration (> 6 months).

**Conclusion:**

The study suggests that VDS could improve insulin concentration and dyslipidemia in the elderly population. The systematic review was registered in Alborz university of medical sciences with 2060-01-03-1397 number and the Ethics council IR.ABZUMS.REC.1397.207 number.

**Supplementary Information:**

The online version contains supplementary material available at 10.1186/s13098-022-00859-0.

## Introduction

'Cardiac-metabolic' is a complex disorder characterized by dyslipidemia, hyperglycemia, visceral obesity, and hypertension [1]. The prevalence of cardiac-metabolic disorders has profoundly grown over the past decade [2, 3]. Individuals with cardiac-metabolic disorders are at a development risk for diabetes mellitus (DM) and cardiovascular disease [4]. In recent years, several studies have investigated the effect of essential nutrients on cardiac-metabolic disorders and their risk factor [5, 6]. As a global health problem, “vitamin D” deficiency is extremely prevalent in all age populations, especially in older adults (≥ 60 years old) [7]. Moreover, there has been growing data to prove the extra-skeletal role of vitamin D [8]. Evidence has been concerned with the relation between vitamin D deficiency and the development of DM, metabolic syndrome, cardiovascular disease, autoimmune diseases, and some cancers [8, 9].

A reverse association has been demonstrated between vitamin D levels and multiple pathological outcomes, including cardiovascular diseases [10, 11]. In this regard, some investigations have suggested that vitamin D supplementation (VDS) has therapeutic results on insulin resistance, serum triglycerides, and waist circumference in patients with metabolic syndrome and even in healthy adults [12–14]. In contrast, a few randomized clinical trials (RCTs) did not show any effectiveness in metabolic profile via VDS [15, 16]. In general, disagreement in the results of studies could be ascribed to differences in terms of methodology, supplementation dose, and populations' characteristics. Moreover, several meta-analyses have examined the impact of VDS on DM, dyslipidemia, body weight, and cardiovascular disease in adults and children and found conflicting results [17–24].

To the best of our knowledge, there is no comprehensive evidence outlining the effects of VDS on cardiac-metabolic outcomes in the elderly population. Given the critical assessment of the impact of vitamin D in older adults, we aimed to examine the overall effects of VDS on cardiac-metabolic outcomes in the elderly population.

## Methods

### Search strategy

In order to assess the relevant documents regarding the association of VDS and cardiac-metabolic outcomes in the elderly population, the biomedical electronic databases, including Scopus, Web of Science, PubMed, EMBASE, Cochrane, and also congress and conference papers as grey literature were searched for all related literature published up to 30 September 2021. Medical Subject Headings (MeSH) terms and Emtree pathways were also used in developing search terms.

The search protocol was developed based on two primary roots of "Vitamin D" and "Elderly". All related components of vitamin D including "25-hydroxyvitamin D", Dihydrotachysterol, "25(OH)D”, Calciol, Calciferol, Cholecalciferol, “25-Hydroxyergocalciferol”, “25-Hydroxycalciferol”, "Ergocalciferol", Calcitriol and elderly including "Older Adult”, Elder, or “Middle-Aged” were considered for searched queries (Additional file 1). There was no restriction for language of publication.

### Inclusion and exclusion criteria

All eligible publications which considered the following criteria were included in this systematic review; (1) trials included elderly participants (participants more than 60 years were considered as elderly in the study) [25, 26], (2) trials administered vitamin D_2_ or vitamin D_3_ (cholecalciferol) as only intervention comparison placebo group, (3) trials reports cardiac-metabolic risk factors as outcomes (lipid profile, glycemic indices, anthropometric measures) (4) studies with clinical trial design, randomized or non-randomized. In addition, we included any dose, duration, and route of intervention. We exclude combination interventions, duplicate citations, narrative and systematic reviews, observational, cross-sectional, and case–control studies, book chapters, and conference papers.

### Validity assessment and data extraction

After three assessment steps for titles, abstracts, and full texts, the full text of each selected record was retrieved for detailed analysis. Data were extracted using a data collection form recording citation, publication year, study year, country, type of study, population characteristics and methodological criteria (sample size, mean age, type of measure), and results and end-points. All proceedings from the comprehensive search to final data extraction were conducted by two independent expert investigators. Probable disagreements between two experts were resolved under the supervision of the primary investigator. The quality of study design, sampling strategy, and risk of bias assessments was conducted according to the Consolidated Standards of Reporting Trials (CONSORT) statement. The Kappa statistic for agreement between two research experts for quality assessment was 0.92. Baseline vitamin D deficiency was certain as serum vitamin D levels < 50 nmol/l, vitamin D insufficiency 50–75 nmol/l, and vitamin D sufficiency > 75 nmol/l [27].

### Statistical analysis

For each cardiac-metabolic outcome, mean changes between baseline and follow-up for each group were compared in the meta-analysis calculation. The Chi-square based 'Q' test and 'I' square statistics were performed to examine the heterogeneity of the studies. The result of the 'Q' test was regarded to be statistically significant at P < 0.1. Standardized mean difference (SMD) was combined using a random-effect model due to heterogeneity between studies [28, 29]. SMD was calculated as [change in outcome/pooled standard deviation of outcome]. Results of the meta-analysis were presented schematically by forest plot. Effect of potential sources of heterogeneity (such as the risk of bias, duration of the intervention (≤ 6 months or > 6 months), intervention dose (< 2000 IU/d or ≥ 2000 IU/d), health condition (type of disease), and baseline vitamin D level (< 50 nmol/l or ≥ 50 nmol/l) were assessed using random-effect meta-regression analysis. Publication bias was estimated by the Egger’s and trim & fill method, which was considered statistically significant at 0.05. The analyses were conducted using STATA 11 software.

### Ethical considerations

The present study was approved by the ethical committee of the Alborz University of Medical Science with IR.ABZUMS.REC.1397.207 number. Whenever we needed more information about a given study, we directly contacted the corresponding author of the cited publications.

## Results

### Study selection

**Fig. 1:**
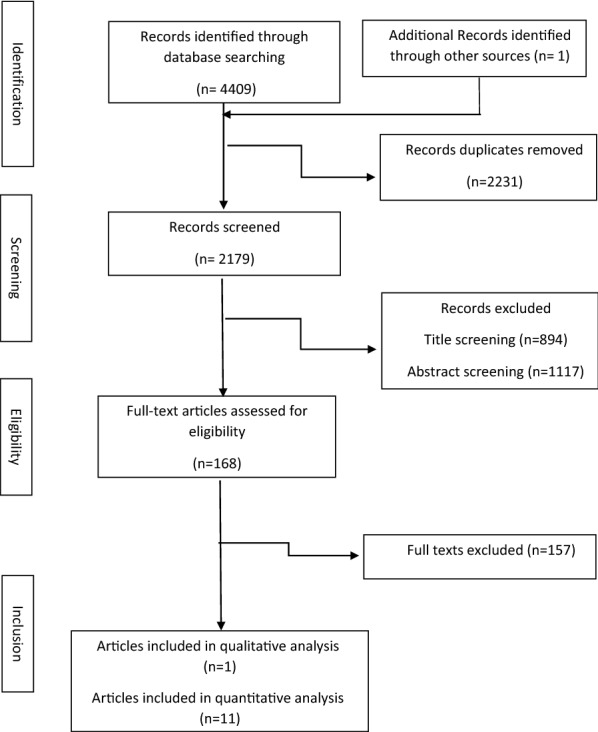
The review flowchart for selection studies

We found 4409 records via a comprehensive search in the review. Out of this number, 2231 documents were identified as duplicates. Two reviewers exactly screened documents by title and abstract, and 2011 records were excluded from the process. Finally, from 168 full texts, 12 eligible trials [13, 30–40] were selected for the systematic review and meta-analysis (Fig. 1).

### Study characteristics and qualitative synthesis

The included trial characteristics are represented in Table 1. The studies were published between 2010 and 2020. Ten of included studies have been designed as RCT [31–40] and ones as non-RCT [30], and Verrusio et al. [13] designed their study as a before-after experiment, so it was not analyzed in the meta-analysis. Six studies were conducted in the European countries [13, 30, 32, 33, 36, 38], two in Lebanon [39, 42], and four enrolled in other countries. The trials included 1328 participants; eleven studies recruited both genders, while one included only women [31]. Participants of five studies suffered from DM [30, 31, 34, 38, 40]. Three studies were conducted on apparently healthy participants [36–38]. Furthermore, other participants had a history of stroke [32], NAFLD [39], and overweight [35], and one study was conducted on postmenopausal women [33]. VDS dosage was varied between 400 IU/day to 4000 IU/day. In two studies, a single oral dose of 100,000 IU or 200,000 IU has been prescribed [30, 32]. The intervention period ranged from 2 months to one year. The included outcome variables were BMI, WC, HbA1c, insulin concentration, FBS, HOMA-IR, TG, Total cholesterol, HDL, LDL, and LDL/HDL Ratio (Table 1).

**Table 1:** Characteristics of included studies

Author, year	Country	Study population	Study design	Sex	Sample size	Dose of Vitamin D (IU)	Mean age (SD)	Intervention duration	Baseline Vitamin D Level (nmol/L) Mean (SD)	outcomes
Witham 2010	UK	DM	RCT	M/F	I_1_: 19 I_2_: 20 P: 22	Two single doses I_1_: 100,000 IU I_2_: 200,000 IU	I_1_:65.3 (11.1) I_2_:63.3 (9.6) P: 66.7 ( 9.7)	–	I_1_:48 (21) I_2_:41(14) P: 45 ( 17)	HOMA-IR HbA1c TC
Naharci 2012	Turkey	IFG	Non-RCT	M/F	I: 28 P: 23	Deficient: 300,000 IU/week Insufficient: 880 IU/day Normal: 400 IU/day	I: 75.1 (7.3) P: 76.1( 5.4)	5 months	I: 47.5 (24.2) P: 55.8 (37.4)	FBS HOMA-IR Insulin TC, TG, LDL, HDL LDL/HDL ratio BMI
Witham 2012	UK	Patient with a history of stroke	RCT	M/F	I: 30 P: 28	A single dose 100,000 IU	I: 66.2 (13) P: 67.7 ( 6.9)	-	I: 38.7 (17.6) P: 38.7 (17.8)	TC
Wood 2012	UK	Postmenopausal women	RCT	F	I_1_:102 I_2_:101 P: 102	I_1_: 400 IU/day I_2_: 1000 IU/day	I_1_: 63.5(1.9) I_2_: 64.1 (2.3) P: 63.9 (2.3)	12 months	I_1_:32.7(12.9) I_2_:32.4(13.8) P: 36.1 (17.1)	FBS HOMA-IR Insulin TC, TG, LDL, HDL
Kim 2014	Korea	DM	RCT	M/F	I: 11 P: 13	1200 IU /day	I: 73.2 (2.0) P: 70 (1.3)	12 weeks	I: 26.1(4.4) P: 29.1 (6.9)	FBS HOMA-IR Insulin TC, TG, LDL, HDL BMI
El-Hajj Fuleihan 2017	Qatar	Overweight	RCT	M/F	High dose(I): 110 Low dose(P): 112	High dose(I): 3750 IU/day Low dose(P): 600 IU/day	T: 71 (4)	12 months	I: 52.1(17.4) P: 49.9(20.4)	FBS HOMA-IR HbA1c, Insulin TC, TG, LDL, HDL
Walter Verrusio, 2017	Italy	Healthy	Before and After	M/F	200	4000 IU/day	T: 65.9 (9.8)	6 months	–	–
Prusik 2018	Poland	Healthy	RCT	M/F	I: 48 P: 31	4000 IU/day	T: 68.4 ( 5.0)	12 weeks	I: 45.1(22.7) P: 60.4(30.2)	TC, TG, LDL, HDL LDL/HDL ratio BMI
El Hajj 2019	Lebanon	Healthy	RCT	M/F	I: 60 P: 55	10,000 IU/week	I: 73.05 (1.95) P:73.56 (2.14)	6 months	I: 25.2 (6.9) P:26.2 (2.14)	BMI WC
Wenclewska 2019	Poland	Healthy & DM	RCT	M/F	I: 48 P: 44	2000 IU / day	I: 63.43(1.5) P: 69.78 (2.1)	3 months	I: 18.2 (3.2) P:18.0(2.1)	FBS HOMA-IR HbA1c TC, TG, LDL, HDL LDL/HDL ratio BMI
Hoseini 2020	Iran	NAFLD	RCT	M/F	I: 10 P: 10	50,000 IU/week	I: 61.3 (1.4) P: 62 (1.8)	8 weeks	I: 22.9 (4.3) P: 24.0 (4.3)	FBS HOMA-IR Insulin TC, TG, LDL, HDL
El Hajj 2020	Lebanon	DM	RCT	M/F	I: 45 P: 43	30,000 IU/week	I: 66.9 (4.1) P: 65.7 (4.5)	6 months	I:14.8 (4.5) P:15.0 (4.2)	FBS HOMA-IR HbA1c TC, TG, LDL, HDL BMI, WC

*RCT* Randomized controlled trial, *I* Intervention, *P* Placebo, *SD* standard deviation, *M* male, *F* female, *DM* Diabetes mellitus, *IFG* Impaired fasting glucose, *NAFLD* Non- alcoholic fatty liver disease, *IU* International unit, *HOMA-IR* Homeostatic Model Assessment of Insulin Resistance, *HbA1c* Hemoglobin A1c, *FBS* Fasting blood sugar, *TC* Total cholesterol, *TG* Triglyceride, *HDL* high-density lipoprotein, *LDL* low-density lipoprotein, *BMI* Body mass index, *WC* Waist circumference

### Quantitative synthesis

We pooled the SMD of cardiac-metabolic outcomes, which were reported through 2 or more studies, using the random-effect meta-analysis procedure owing to high heterogeneity among the included studies. The results of the (I^2^) test and estimated P-value are displayed in Table 2.

**Table 2:** Meta-analysis of the effect of vitamin D supplementation on cardiac-metabolic outcomes in elderly population

Variables	Number of study	Pooled SMD ( 95% CI)	Model	Heterogeneity assessment		
				I^2^	Q test	P-value
HbA1c	4	0.06(− 0.11, 0.24)	Random	0.0	0.57	0.96
FBS (mg/dl)	7	0.01(− 0.2, 0.21)	Random	52.5	14.75	0.04
HOMA-IR	8	0.0(− 0.14, 0.13)	Random	6	9.57	0.38
Insulin (μU/ml)	5	− 0.11(− 0.34, 0.14)	Random	54.4	10.97	0.05
TG (mg/dl)	8	− 0.45(− 0.86, − 0.04)	Random	88.5	69.46	0.0
TC (mg/dl)	10	− 0.14(− 0.25, − 0.02)	Random	0.0	10.49	0.48
HDL (mg/dl)	8	0.34(− 0.07, 0.74)	Random	88.6	69.91	0.0
LDL (mg/dl)	8	− 0.17(− 0.36, 0.02)	Random	47.9	15.37	0.05
LDL/HDL Ratio	3	− 0.08(− 0.35, 0.19)	Random	0.0	0.14	0.93
BMI (kg/m^2^)	6	0.18(− 0.3,0.67)	Random	83.7	30.72	0.0
WC(cm)	2	− 0.24(− 0.98, 0.24)	Random	85.3	6.80	0.0

### The effect on lipid profile

**Fig. 2:**
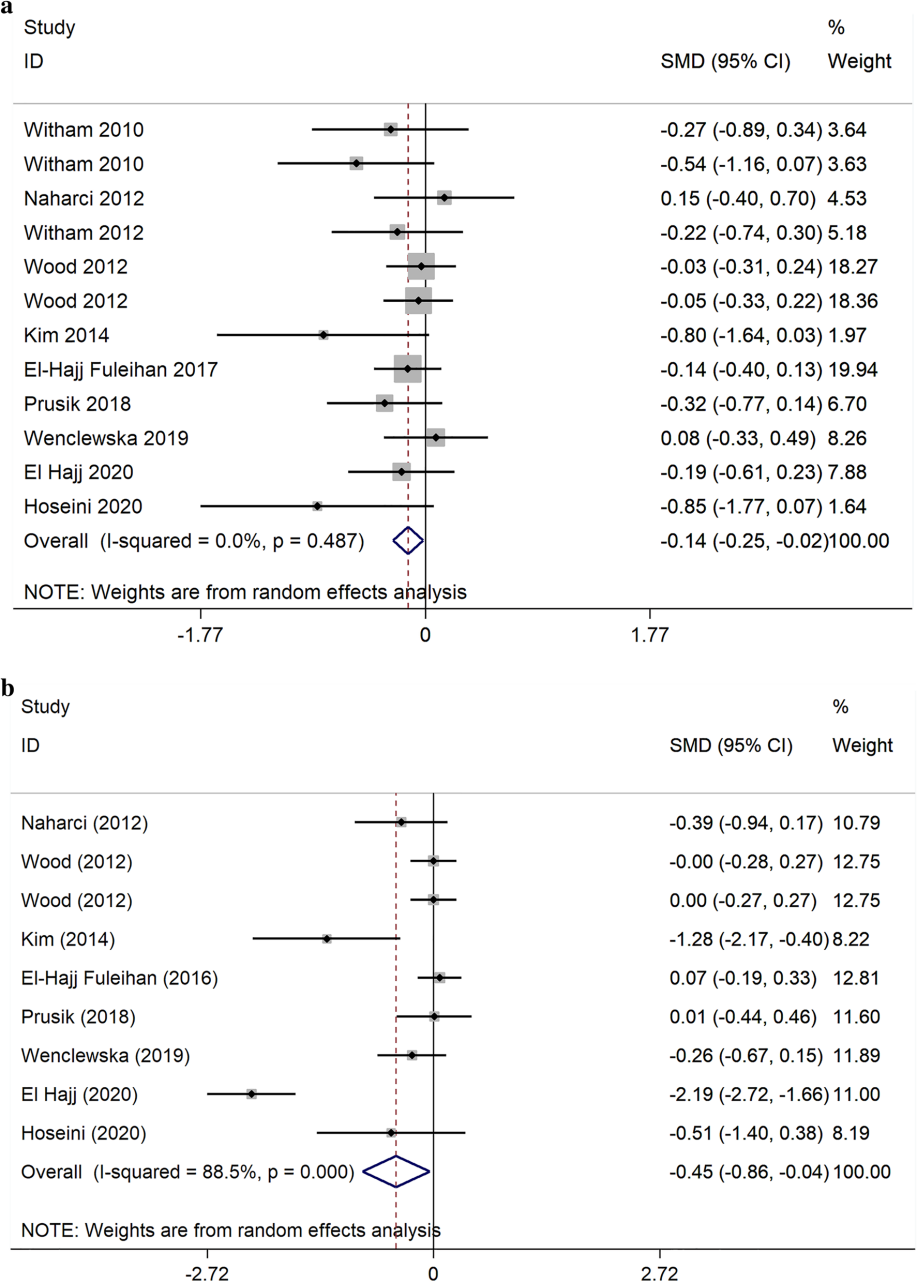
Forest plot of the effect of vitamin D supplementation on **a** total cholesterol, **b** triglyceride

Ten trials [30–36, 38–40] reported the VDS effects on TC. Pooling the SMD revealed significant reduction in TC following intervention compared to placebo (SMD: − 0.14 mg/dl; 95% CI: − 0.25, − 0.02), without any significant heterogeneity (I^2^ = 0; P = 0.48) (Fig. 2). Also, changes in TG was assessed in 8 studies. Compared with placebo, the difference in TG decrease was significant, (SMD: − 0.45 mg/dl; 95%CI: − 0.86, − 0.04). The heterogeneity of the analysis was high between studies (I^2^ = 88.5%; P = 0.00) (Fig. 2). The pooled SMD for LDL (SMD: − 0.45 mg/dl; 95%CI: − 0.88, − 0.04), HDL (SMD: 0.34 mg/dl; 95%CI: − 0.07, 0.74) and LDL/HDL Ratio (SMD: − 0.08 mg/dl; 95%CI: − 0.35, 0.19) showed a non-significant change in the VDS group in comparison with the placebo (Additional file 2.

**Fig. 3:**
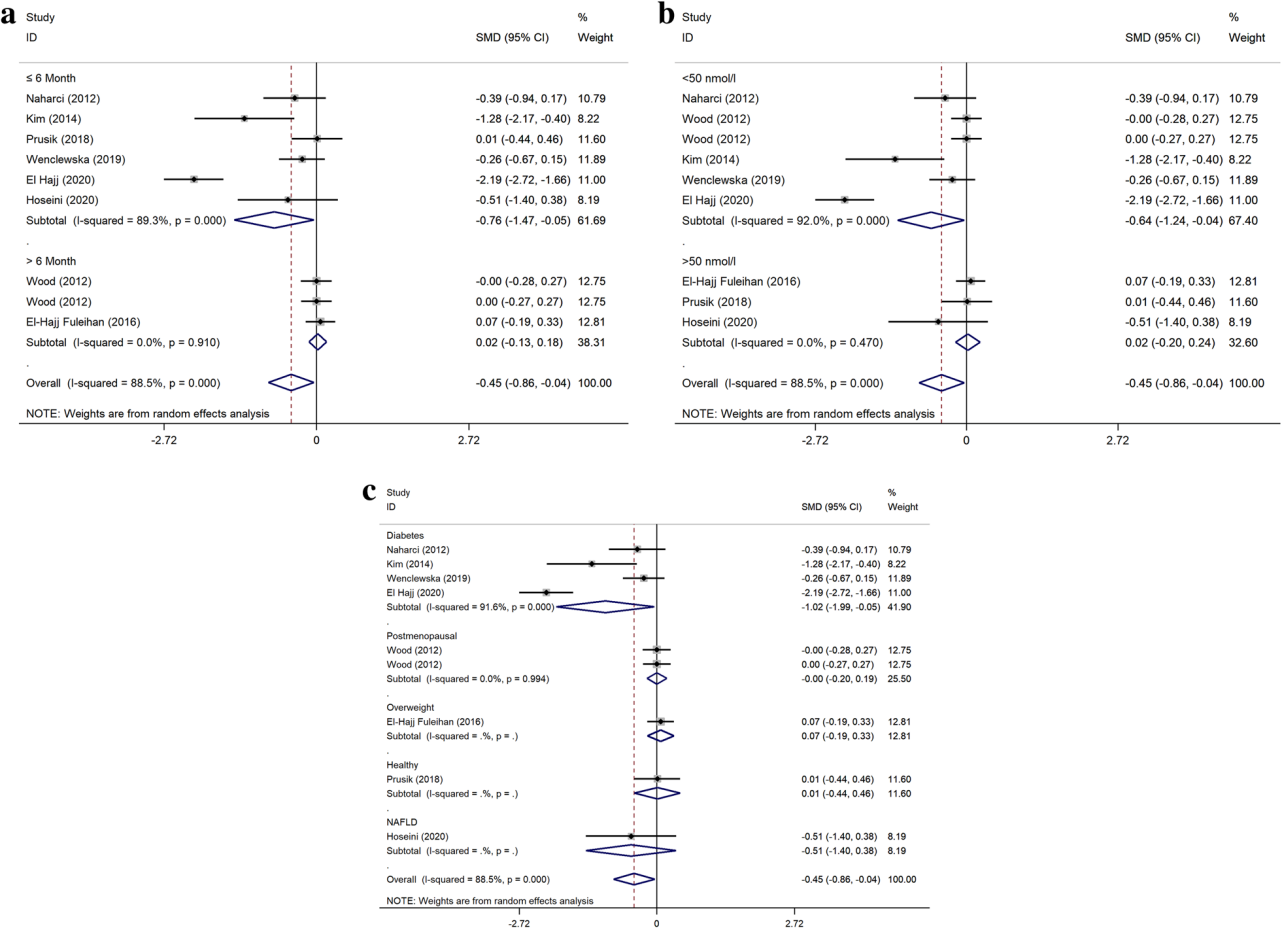
Forest plot of the effect of vitamin D supplementation on triglyceride (mg/dL) stratified by intervention duration (**a**) stratified by baseline vitamin D levels (**b**) stratified by disease subtype (**c**)

The subgroup analysis finding as regards the risk of bias of selected articles, baseline vitamin D, diseases, dose, and duration of intervention revealed a significant effect of VDS in the reduction of TG concentrations in the population with vitamin D deficiency, DM, and study duration ≤ 6 months subgroup (Fig. 3). The subgroup analysis did not statistically meaningful effect concerning other lipid profile outcomes (data was not shown).

### The effect on glycemic indices

**Fig. 4:**
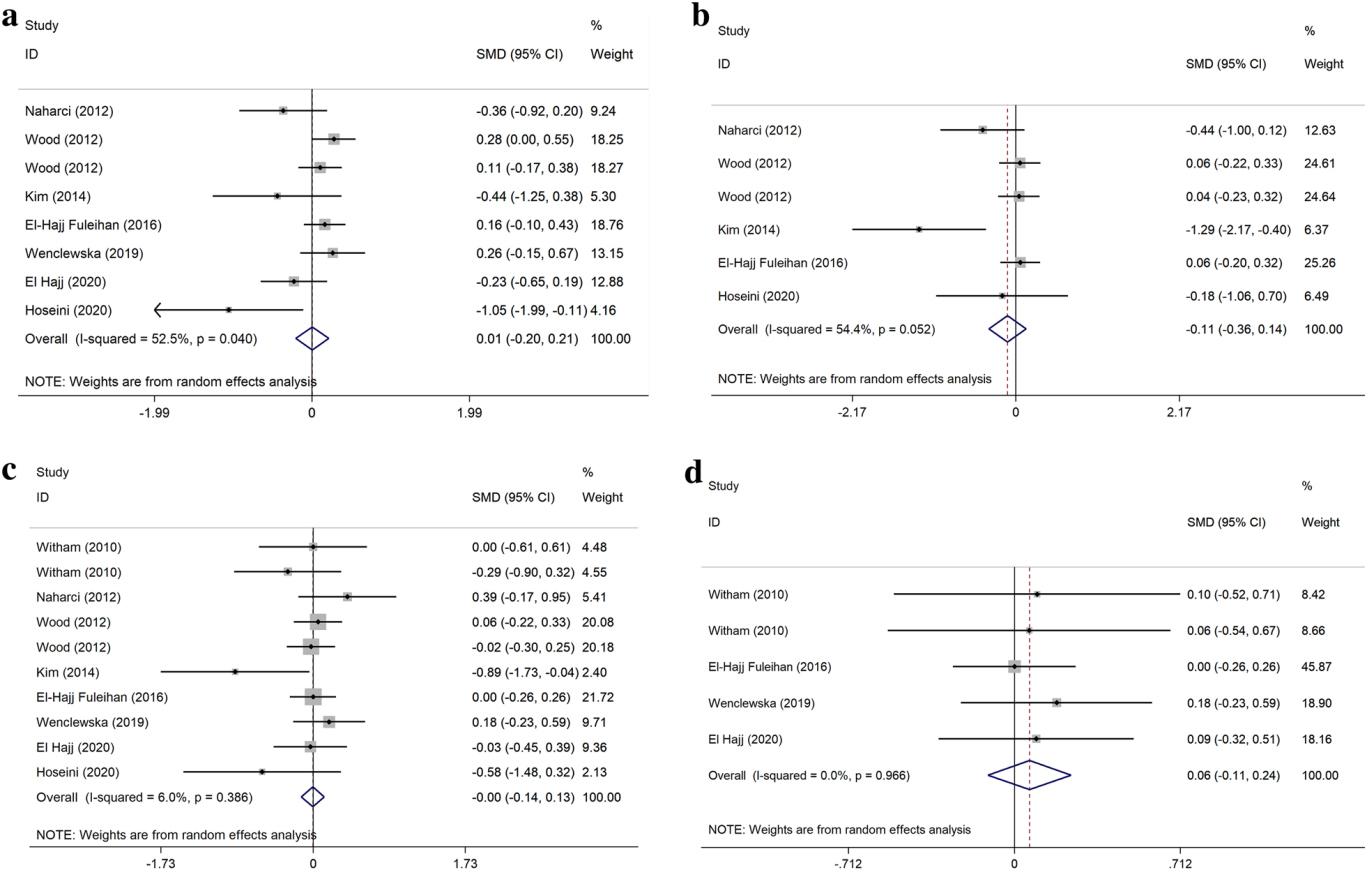
Forest plot of the effect of vitamin D supplementation on fasting blood sager (**a**), insulin concentration (**b**), Homeostatic Model Assessment of Insulin Resistance (**c**), Hemoglobin A1c (**d**)

The pooled SMD of five studies [31, 33–35, 39] indicated a decreased in insulin concentration in vitamin D group, however it was not statistically significant (SMD = − 0.11 μU/ml; 95% CI: − 0.34, 0.14), with moderate heterogeneity (I^2^ = 54.4% P = 0.05). Additionally, Pooled the effects of VDS on FBS, HOMA-IR, and HbA1c compared to the placebo was (SMD: 0.01 mg/dl; 95% CI: − 0.2, 0.21), (SMD: 0.0;95% CI: − 0.14, 0.13), and (SMD:0.06;95% CI: − 0.11, 0.24) respectively (Fig. 4). The results of subgroup analysis were demonstrated a significant effect in insulin concentration in study duration ≤ 6 months (SMD: − 0.6 μU/ml; 95% CI: − 1.18, − 0.02), (I^2^ = 42.0% P = 0.17) (Additional file 4). However, FBS levels in study duration > 6 months significantly increased (SMD: 0.18 mg/dl; 95% CI: 0.02, 0.34), (I^2^ = 0.0% P = 0.67) (Additional file 3).

### The effect on anthropometric measures

Six studies measured the effect of VDS on BMI [30, 34, 36–38, 40]. Overall, the meta-analysis revealed no difference in BMI between the study groups (SMD: 0.18 kg/m^2^; 95% CI: − 0.30, 0.67); results showed high heterogeneity (I^2^ = 83.7% P = 0.00) (Additional file 4). Furthermore, VDS did not change WC compared with placebo (SMD: − 0.24 Cm; 95% CI: − 0.98, 0.49), (I^2^ = 85.3% P = 0.00) (Additional file 4). The subgroup analyzed based on the risk of bias, diseases, and vitamin D dose didn't show any notable changes in the overall SMD of BMI (P > 0.05) (data was not shown).

### Risk of bias assessment

The risk of bias assessments of the selected RCTs is shown in Table 3. Two studies had CONSORT score equal to 33, which is interpreted as high quality. Six trials were labeled as medium quality according to CONSORT scores (range: 25–30). The score of the three studies was lower than 25 and was classified as low quality. Random allocation and concealment mechanisms and blinding details were the main sources of risk of bias in the included trials.

**Table 3:** Risk of bias assessment of included studies according to the CONSORT Checklist

Item	Cynthia El Hajj, 2019	Cynthia El Hajj, 2020	Ghada El-Hajj Fuleihan, 2017	Zahra Hoseini, 2020	Ilkin Naharci, 2012	Kim, Hyoung-Jun, 2014	Krzysztof Prusik, 2018	Sylwia Wenclewska, 2019	M. D. Witham, 2010	M.D. Witham, 2012	Adrian D. Wood, 2012
1a	Yes	Yes	Yes	Yes	No	No	Yes	Yes	Yes	Yes	Yes
1b	Yes	Yes	Yes	Yes	Yes	Yes	Yes	Yes	Yes	Yes	Yes
2a	Yes	Yes	Yes	Yes	Yes	No	Yes	Yes	Yes	Yes	Yes
2b	Yes	Yes	Yes	Yes	Yes	Yes	Yes	No	Yes	Yes	No
3a	Yes	Yes	Yes	Yes	Yes	Yes	Yes	Yes	Yes	Yes	Yes
3b	No	No	No	No	No	No	No	No	No	No	No
4a	Yes	Yes	Yes	Yes	Yes	Yes	Yes	Yes	Yes	Yes	Yes
4b	Yes	Yes	Yes	Yes	Yes	Yes	Yes	Yes	Yes	Yes	Yes
5	Yes	Yes	Yes	Yes	Yes	Yes	Yes	Yes	Yes	Yes	Yes
6a	Yes	Yes	Yes	Yes	Yes	Yes	Yes	Yes	Yes	Yes	Yes
6b	No	No	No	No	No	No	No	No	Yes	No	No
7a	Yes	Yes	Yes	No	Yes	Yes	Yes	Yes	Yes	Yes	Yes
7b	Yes	Yes	Yes	Yes	Yes	Yes	Yes	Yes	Yes	Yes	Yes
8a	Yes	Yes	Yes	Yes	No	Yes	Yes	Yes	Yes	Yes	Yes
8b	No	No	No	No	No	Yes	Yes	Yes	No	Yes	Yes
9	Yes	Yes	No	No	No	Yes	Yes	Yes	Yes	Yes	Yes
10	Yes	Yes	No	No	No	Yes	No	No	No	Yes	Yes
11a	Yes	Yes	No	No	No	No	No	No	No	Yes	Yes
11b	No	No	No	No	No	No	No	No	No	Yes	Yes
12a	Yes	Yes	Yes	Yes	Yes	Yes	Yes	Yes	Yes	Yes	Yes
12b	Yes	No	No	No	No	No	No	Yes	No	Yes	Yes
13a	Yes	Yes	Yes	Yes	No	No	No	No	Yes	Yes	Yes
13b	Yes	Yes	Yes	Yes	No	No	No	No	Yes	Yes	Yes
14a	Yes	Yes	Yes	Yes	Yes	Yes	Yes	Yes	Yes	Yes	Yes
14b	No	No	No	No	No	No	No	No	No	No	Yes
15	Yes	Yes	Yes	Yes	Yes	Yes	Yes	Yes	Yes	Yes	Yes
16	Yes	Yes	Yes	Yes	Yes	Yes	Yes	Yes	Yes	Yes	Yes
17a	Yes	Yes	Yes	Yes	Yes	Yes	Yes	Yes	Yes	Yes	Yes
17b	Yes	Yes	No	Yes	No	No	No	Yes	No	Yes	Yes
18	No	No	Yes	No	Yes	No	No	No	Yes	Yes	No
19	No	No	Yes	No	Yes	Yes	Yes	Yes	Yes	No	Yes
20	Yes	Yes	Yes	Yes	Yes	Yes	Yes	Yes	Yes	Yes	Yes
21	Yes	Yes	Yes	Yes	Yes	Yes	Yes	Yes	Yes	Yes	Yes
22	Yes	Yes	Yes	Yes	Yes	Yes	Yes	Yes	Yes	Yes	Yes
23	Yes	Yes	Yes	Yes	No	No	Yes	Yes	Yes	Yes	Yes
24	Yes	Yes	No	Yes	No	No	No	No	Yes	Yes	Yes
25	Yes	Yes	Yes	Yes	Yes	Yes	Yes	Yes	Yes	Yes	Yes
Total	30	29	26	23	21	23	25	26	29	33	33

### Publication bias and sensitivity analysis

Publication bias was performed by Egger's test. According of Egger’s test results the publication bias was seen by VDS on TC (coefficient = − 1.69, standard error = 0.59, P = 0.018, 95% CI = − 3.02, − 0.36) and FBS (coefficient = − 3.02, standard error = 0.77, P = 0.008, 95% CI = − 4.91, − 1.14). Because of the evidence of publication bias, "trim-and-fill" analysis was conducted to estimate potentially missing trials. The result of "trim-and-fill" presented no missing trials, and the publication bias has an ignorable effect on pooled SMD of TG and FBS. There was no evidence of publication bias on the impact of vitamin D with other studies' outcomes (Egger's P > 0.05). The results of the sensitivity analyses, based on the one-out remove method, showed that there was no substantial difference in the effect sizes of any of the pooled results (Data was not shown).

## Discussion

The present meta-analysis finding revealed that VDS compared with placebo improved TC and TG management in the elderly. In contrast, we observed no significant beneficial effects of VDS in overall or subgroup analyses on LDL, HDL, FBS, HbA1c, HOMA-IR, BMI, and WC. Nevertheless, characteristics of the selected trials such as baseline vitamin D, duration of supplementation, and health condition of participants partly influenced the impacts that emerged in some overall findings.

Given the existence of much evidence about the effectiveness of vitamin D in glucose metabolism, some meta-analyses similar to our overall pooled results found that VDS may not improve glycemic indices[23, 41]. While, others [22, 42, 43] proposed that vitamin D could be a useful adjuvant therapy in controlling glycemic parameters in patients with DM or impaired glucose tolerance because it reduces insulin resistance and improves the function of beta cells [44]. Most systematic reviews have evaluated the effects of vitamin D on glycemic parameters in diabetics or adults with impaired glucose tolerance. Our selection criteria were different from the meta-analysis, and we included the elderly population (more than 60 years) with any type of disease. Despite that, the subgroup analysis showed a decrease in glycemic parameters (i.e., insulin concentration, FBS, and HOMA-IR) in DM subgroups; but, because of the few eligible trials, it is not statistically significant.

Further, in terms of the intervention duration, present findings demonstrated that intervention duration ≤ six months significantly improved insulin concentration; however, long-term duration (> 6 months) was associated with increased FBS levels. In line with our findings, some meta-analyses displayed improvement in glycemic control in short-term interventions [22, 41, 45]. Contrary to the results, some studies found that longer intervention durations were associated with more decrease in glycemic parameters [20, 46, 47]. It seems that the effect of the duration of VDS on glycemic control is controversial. This ambiguity may be attributed to the wide diversity between the study's population, health condition, dose, frequency, and baseline vitamin D level.

Concerning lipid profile, our findings agree with the results of most previous meta-analyses revealed that vitamin D could be a valuable treatment to decrease TC and TG. In addition, a significant reduction was shown in TG in the short-term intervention (≤ 6 months) studies, patients with DM and low levels of baseline vitamin D. Dibaba et al. (2019), in a comprehensive meta-analysis found administration of vitamin D diminish TC, TG, and LDL cholesterol, while the improvements were more noticeable in patients with vitamin D deficiency [18]. Another meta-analysis reported that treatment with vitamin D has a beneficial impact on TC; lowering LDL in diabetics and patients with insufficient vitamin D and shorter duration was significant. However, there was no positive effect on TG and HDL cholesterol [48]. In Mirhosseini et al. meta-analysis vitamin D administration improved cardiac-metabolic parameters such as lipid profile. Also, the improvement effect of vitamin D was noticeable in trials with less than six months of intervention and patients with baseline serum vitamin D levels < 50 nmol/l [22]. Wang et al. provided that vitamin D changed LDL cholesterol but not TG, TC, and HDL cholesterol. As well as, the effect on LDL-C was more evident in the short-term intervention duration [49]. However, a recent meta-analysis of 4 RCTs that evaluate the impact of vitamin D on lipid profile in adults with metabolic syndrome has shown no significant effect on lipid profile following supplementation [17]. In the present study, probably due to insufficient studies, no statistically meaningful change was seen in LDL and HDL cholesterol.

It is considered that an inverse association was seen between serum vitamin D levels and serum lipid profile [10, 50]. Several possible reasons may explain the role of vitamin D in modifying lipid profiles. Vitamin D might increase lipoprotein lipase activity and mRNA in adipocytes and lower serum TG levels [51, 52]. Also, it decreases the absorption of fatty acid via improving calcium absorption, and the formation of insoluble calcium-fatty acid complexes in the bowel leading to a diminution cholesterol concentration [53].

There were masses of evidence outlining the emphasis of vitamin D on human health and diseases [54, 55]. Many studies have revealed that impaired vitamin D levels may result in multiple pathological outcomes, including obesity, hypertension, cardiovascular diseases, and DM [56, 57]. Studies have demonstrated that older papulation are highly at risk of severe vitamin D deficiency [58]. The effectiveness of vitamin D in preventing chronic diseases, including metabolic syndrome and cardiovascular diseases, has received extra attention in recent years [54, 59]. Several studies suggested that vitamin D deficiency or insufficiency increases the risk of all-cause mortality in elderly participants, even before the onset of DM and cardiovascular diseases [60]. The result of a meta-analysis demonstrated that serum vitamin D levels of 75 to 87.5 nmol/L (or 30 to 35 ng/mL) are associated with the lowest mortality rate [61]. Taking into expanding the elderly population in the world, the importance of chronic disease and its complications in the population increases. Therefore, as a cheap and safe treatment, vitamin D might prevent morbidities and fracture events in the elderly [62]. As mentioned above, in our study, vitamin D administration has a therapeutic effect on some cardiac-metabolic parameters, though no beneficial effect was observed on BMI or WC. The inverse relationship between BMI and vitamin D status has been provided in numerous pieces of evidence [13, 63]. However, the vitamin D effects on anthropometric measures are controversial. A recent meta-analysis indicates no significant impact on the BMI and WC of healthy adults [64]. With regard to that, a few RCTs investigated vitamin D impacts on anthropometric measures in the elderly.

This is the first systematic review examining the VDS effects on cardiac-metabolic outcomes in the elderly population (≥ 60 years) to the best of our knowledge. We conducted a comprehensive literature search that included all possible related eligible studies. Also, Subgroup and sensitivity analysis was executed to mitigate the study's heterogeneity. Finally, there was no evidence of significant publication bias in most of the study's outcomes, except in TC and FBS. After using Trim and fill method, the overall effect size of vitamin D on TG remained unchanged. The study has some limitations; First, we found only 11 eligible studies in this meta-analysis, clearly underlining the lack of adequate investigation of the elderly population in this issue. Second, even after performance subgroup analysis, we could not diminish potential heterogeneity in some outcomes such as TG. The third limitation is that data on WC and BMI was available in few studies, thus precluding evaluation of the role of vitamin D on anthropometric measures.

## Conclusion

This study suggests that VDS may improve TC, TG, and insulin concentration in the elderly, especially in short-term intervention duration and in patients with vitamin D deficiency and diabetes. Although the number of studies on this issue is minimal, nonetheless, the finding of this study will enhance our understanding of the importance of vitamin D in the elderly population as well as potentially provide the best evidence-based practical interventions to improve vitamin D-related cardiac-metabolic diseases in the elderly people. Due to, the high heterogeneity between included studies the study results must be interpreted with caution, and large and well-designed RCTs are necessary to validate the review findings.

## Supplementary Information

**Additional file 1** Search strategy

**Additional file 2**. Forest plot of the effect of vitamin D supplementation on high-density lipoprotein; HDL (a) low-density lipoprotein; LDL (b) LDL/HDL ratio (c)

**Additional file3**. Forest plot of the effect of vitamin D supplementation on insulin concentration stratified by intervention duration (a) Forest plot of the effect of vitamin D supplementation on fasting blood sager stratified by intervention duration (b).

**Additional file 4**. Forest plot of the effect of vitamin D supplementation on body mass index (BMI) (a) Waist circumference (b).

## Data Availability

The datasets generated or analyzed during the current study are available from the corresponding author on reasonable request.

## Abbreviations

BMI: Body mass index
CI: Confidence interval
CONSORT: Consolidated Standards of Reporting Trials
DM: Diabetes mellitus
FBS: Fasting blood sugar
HbA1c: Hemoglobin A1c
HDL: High-density lipoprotein
HOMA-IR: Homeostatic Model Assessment of Insulin Resistance
LDL: Low-density lipoprotein
MESH: Medical Subject Headings
RCT: Randomized clinical trial
SMD: Standardized mean difference
TC: Total cholesterol
TG: Triglyceride
VDS: Vitamin D supplementation
WC: Waist circumference
