# Supplementary material for: Effect of vitamin D supplementation on cardiac-metabolic risk factors in elderly: a systematic review and meta-analysis of clinical trials
Source: Diabetol Metab Syndr. 2022 Jun 25;14:88. doi: 10.1186/s13098-022-00859-0 (PMC9233853; doi:10.1186/s13098-022-00859-0)
Supplement: Supplementary file 1 — Additional file 1 Search strategy [file 13098_2022_859_MOESM1_ESM.pdf]

Search strategy

PubMed

("Vitamin D"[Mesh] OR "Vitamin D"[Ti] OR "25-hydroxyvitamin D"[Ti] OR Dihydrotachysterol[Ti] OR "25(OH)D"[Ti] OR Calciol[ti] OR Calciferol[Ti] OR "Vitamin D3"[Ti] OR Cholecalciferol[Ti] OR "25-Hydroxyergocalciferol"[Ti] OR "25-Hydroxycalciferol"[Ti] OR "Ergocalciferol" [Ti] OR Calcitriol[Ti] OR "25(OH)-vitamin D"[Ti] OR "25 (OH) vitamin D"[Ti] OR "1,25(OH)2-vitamin D"[Ti] OR "24,25(OH)-vitamin D"[Ti] OR "25-Hydroxyvitamin D 2"[Ti] OR "24,25-Dihydroxyvitamin D 3"[Ti] OR Dihydroxycholecalciferol[Ti] OR Hydroxycholecalciferol[Ti]) AND ("Older Adult"[tiab] OR elderly[tiab] OR Elder[tiab] OR "Middle Aged"[tiab]) AND 1950/01/01:2021/9/30[dp]

List of excluded studies along with reasons for exclusion:

1) **The studies were not clinical trials**
(Andersen, et al. 2021; Andreeva, et al. 2014; Beck and Ovesen 1999; Brenner, et al. 2017; Breysse, et al. 2015; Cheng, et al. 2015; Del Ser, et al. 2019; Espino, et al. 2010; Fantino, et al. 2011; Fernández-Barrés, et al. 2016; Goh, et al. 2014; Hashemi, et al. 2014; Hribar, et al. 2021; Janssen, et al. 2013; Jungert, et al. 2014; Kauppi, et al. 2013; Larsen, et al. 2005; Lilliu, et al. 2003; Malafarina, et al. 2013; Meunier 1998; Motomura, et al. 1996; Neelemaat, et al. 2010; Oh, et al. 2015; Okubo, et al. 2014; Orces and Gavilánez 2020; Ruwanpathirana, et al. 2014; Sakurai, et al. 2014; Segal, et al. 2004; Shen, et al. 2021; Tizaoui, et al. 2014; Toribio, et al. 2021; Toss, et al. 1982; Vanderschueren, et al. 2013; Welsh, et al. 2012)
2) **Being irrelevant to the main outcomes**
(Abderhalden, et al. 2020; Ackermann and Toro 1953; Alavi, et al. 2019; Aloia, et al. 2019; Aloia, et al. 2005; Aloia, et al. 2018; Aoki, et al. 2018; Aspray, et al. 2019; Azimzadeh, et al. 2020; Bischoff-Ferrari, et al. 2020; Bogaerts, et al. 2011; Borecka, et al. 2021; Bray, et al. 2018; Briguglio, et al. 2020; Broe, et al. 2007; Burns and Paterson 1985; Camargo, et al. 2020; Carvalho, et al. 2018; Chel, et al. 2008; Cuellar, et al. 2019; de Koning, et al. 2019; de Medeiros Cavalcante, et al. 2015; Del Ser, et al. 2019; Dhesi, et al. 2004; Drinka, et al. 2006; Dukas, et al. 2005a; Dukas, et al. 2005b; El Sabeh, et al. 2021; Forbes 2003; Gallagher, et al. 2013; GHOSH, et al. 2021; Goswami, et al. 2012; Grootswagers, et al. 2019; Group 2005; Guerro Prado, et al. 2017; Hamdy, et al. 1987; Janssen, et al. 2010; Jensen, et al. 1982; Jia, et al. 2019; Kamble, et al. 2020; Kooienga, et al. 2009; Latham, et al. 2003; Law, et al. 2006; Martineau, et al. 2015; Moreira-Pfrimer, et al. 2009; Murakami, et al. 2009; Neves, et al. 2006; Papanicolaou, et al. 2013; Pfeifer, et al. 2000; Pirotta, et al. 2015; Prince, et al. 2008; Rahme, et al. 2017; Ranathunga, et al.

2019; Recker, et al. 1999; Renerts, et al. 2019; Rist, et al. 2021; Rodríguez, et al. 2010; Romagnoli, et al. 2008; Rondanelli, et al. 2016; Sato, et al. 2005; Schietzel, et al. 2019; Schild, et al. 2015; Segal, et al. 2009; Shea, et al. 2019; Smith, et al. 2017; Vaes, et al. 2018; Verschueren, et al. 2011; von Restorff, et al. 2009; Wu, et al. 2021; Xia, et al. 2009; Yang, et al. 2018; Zajac, et al. 2020; Zhu, et al. 2008)

3) **Combination therapy of vitamin D + other vitamins or minerals**
(Abe, et al. 2016a; Abe, et al. 2017a; Abe, et al. 2016b; Bauchner and Redberg 2016; Berendsen, et al. 2013; Bo, et al. 2019; Bonjour, et al. 2013; Brazier, et al. 2005; Earthy and da Silva 2008; Foulkes, et al. 2021; Gonçalves, et al. 2020; Groenendijk, et al. 2020; Harwood, et al. 2004; Hidalgo 2013; Himeno, et al. 2009; Janssen, et al. 2013; Kheyruri, et al. 2021; LaCroix, et al. 2009; Liberman, et al. 2019; Miller, et al. 2021; Molnár, et al. 2016; Moran, et al. 2018; Murakami, et al. 2009; Neelemaat, et al. 2012; Pfeifer, et al. 2009; Rathmacher, et al. 2020; Rossom, et al. 2012; Setiati, et al. 2018)

4) **Studying populations of different ages (<60 years)**
(Angellotti, et al. 2019; Caillet, et al. 2013; Canguven, et al. 2017; Cheng, et al. 2013; Ferreira, et al. 2020; Gagnon, et al. 2014; Gonçalves, et al. 2020; Karefylakis, et al. 2018; Kim, et al. 2020; Lemieux, et al. 2019; Lerchbaum, et al. 2019; Lu, et al. 2018; Mozaffari, et al. 2021; Nagpal, et al. 2009; Patti, et al. 2019; Poloni, et al. 2019; Prithiani, et al. 2021; Yin, et al. 2016)

5) **Presented in other included documents**
(2017; Abe, et al. 2017b; Abiri, et al. 2020; Aloia, et al. 2019; Avenell, et al. 2007; Bauer, et al. 2015; Dawson-Hughes, et al. 1991; Hoseini, et al. 2020; Lemieux, et al. 2018; Pfeifer, et al. 2001)
