# Supplementary figures and images for: Effect of vitamin D supplementation on cardiac-metabolic risk factors in elderly: a systematic review and meta-analysis of clinical trials
Source: Diabetol Metab Syndr. 2022 Jun 25;14:88. doi: 10.1186/s13098-022-00859-0 (PMC9233853; doi:10.1186/s13098-022-00859-0)

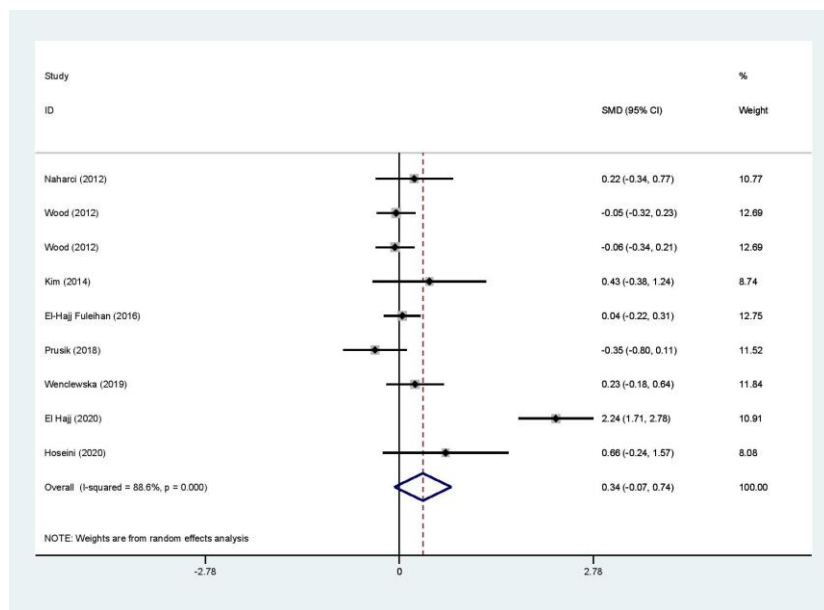

a

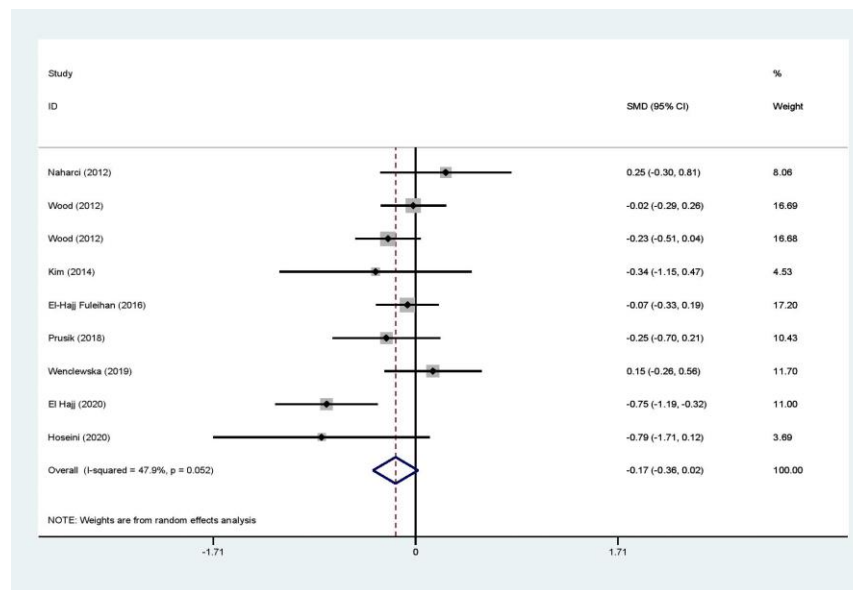

b

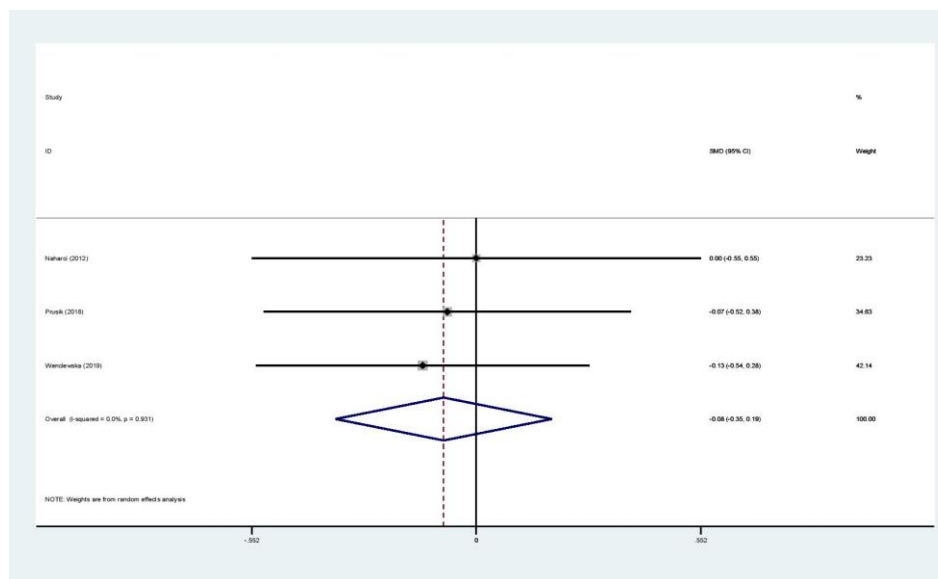

c

Supplement: Supplementary file 2 — Additional file 2. Forest plot of the effect of vitamin D supplementation on high-density lipoprotein; HDL (a) low-density lipoprotein; LDL (b) LDL/HDL ratio (c) [file 13098_2022_859_MOESM2_ESM.pdf]

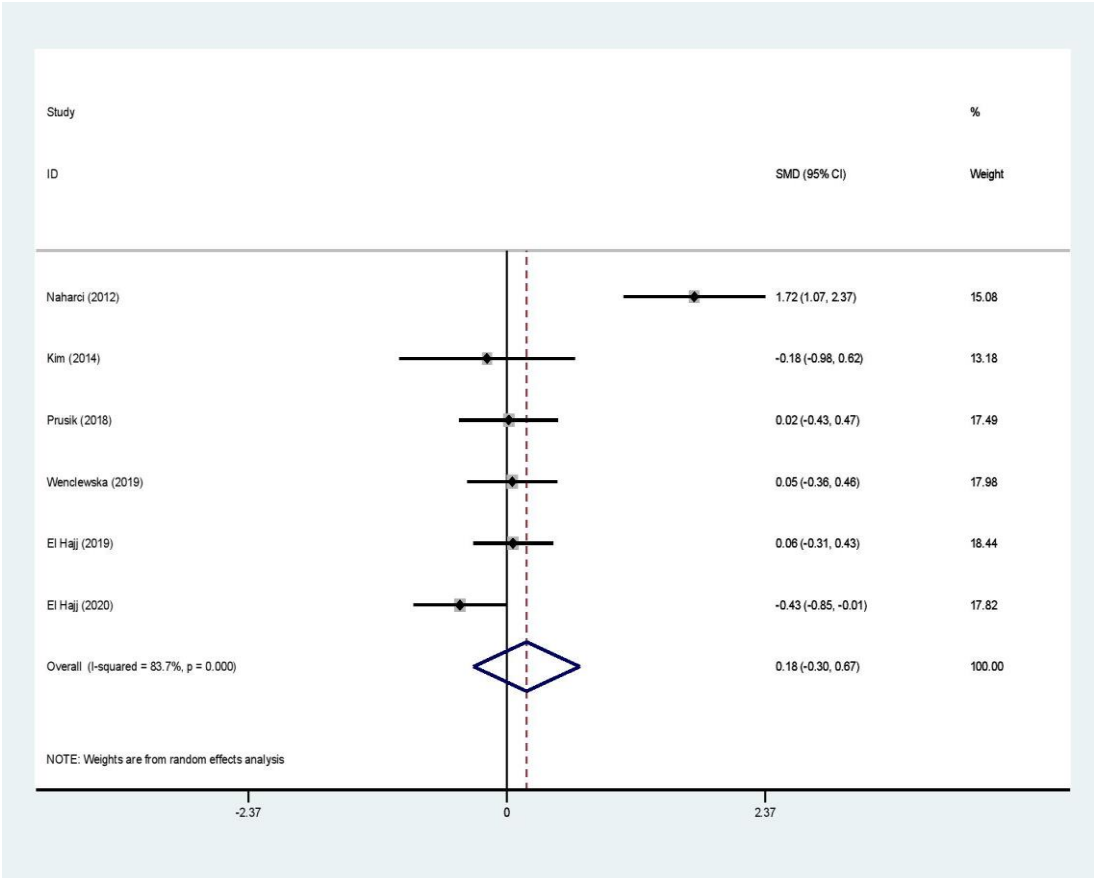

a

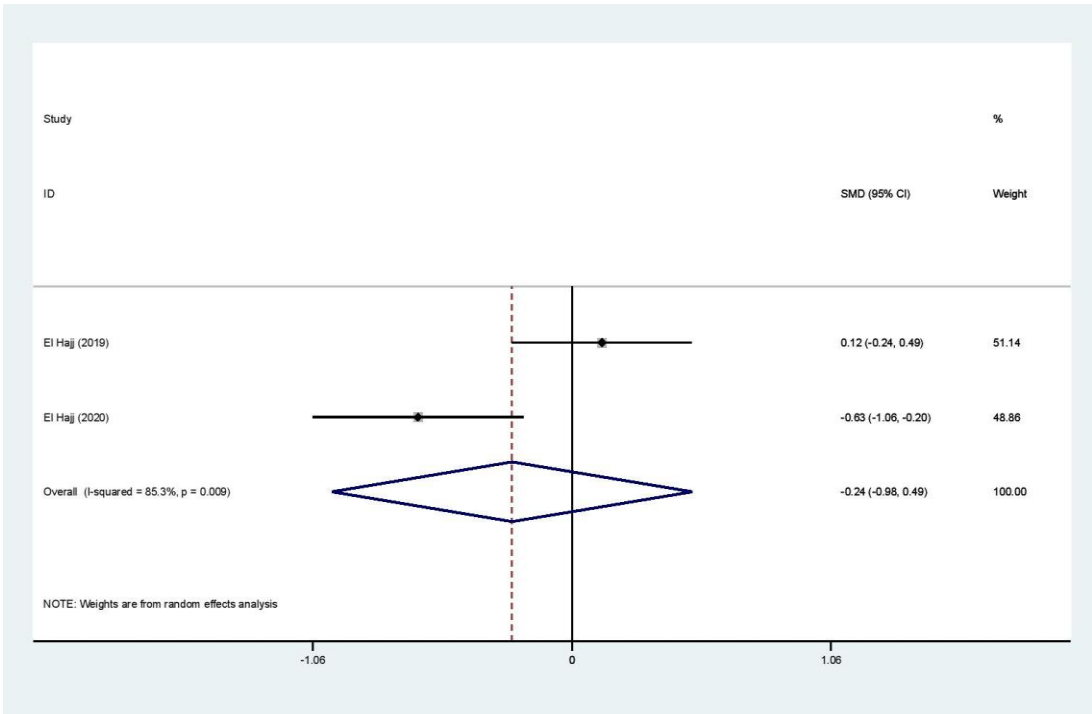

b

Supplement: Supplementary file 3 — Additional file3. Forest plot of the effect of vitamin D supplementation on insulin concentration stratified by intervention duration (a) Forest plot of the effect of vitamin D supplementation on fasting blood sager stratified by intervention duration (b). [file 13098_2022_859_MOESM3_ESM.pdf]

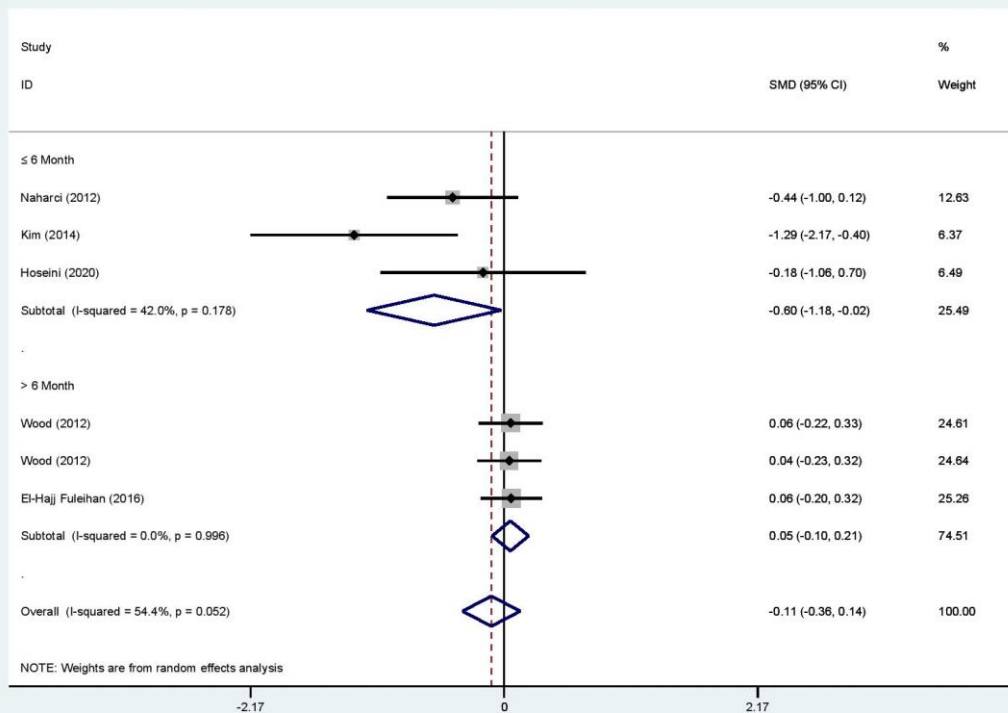

**a**

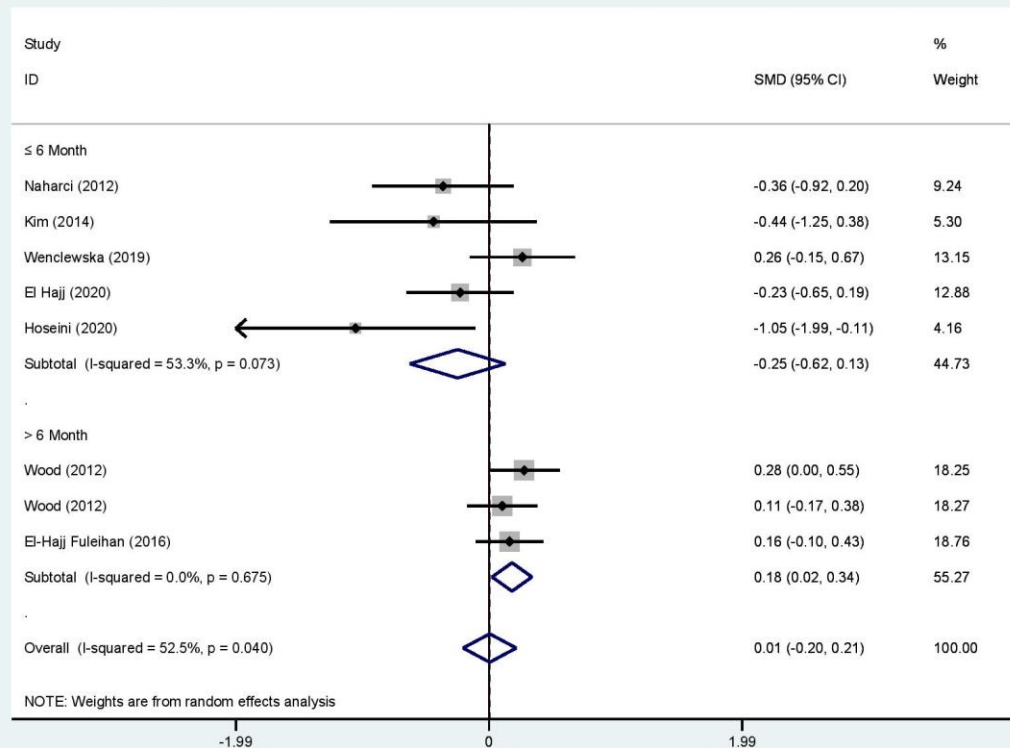

**b**

Supplement: Supplementary file 4 — Additional file 4. Forest plot of the effect of vitamin D supplementation on body mass index (BMI) (a) Waist circumference (b). [file 13098_2022_859_MOESM4_ESM.pdf]
